# 

**Published:** 1874-02

**Authors:** 


Vol. XIII.] FEBRUARY, 1874. [No. 7.

### BUFFALO

### Medical and Surgical Journal.

EDITED BY JULIUS F. MINER, M. D.

Professor of Special Surgery in the Buffalo Medical College; Surgeon to the Buffalo General Hospital; Surgeon to Buffalo Hospital of the Sisters of Charity, etc., etc.

BUFFALO.
PUBLISHED FOR THE EDITOR.

1874.

